# Community perceptions of postmortem examination and minimally invasive tissue sampling in neonates:a qualitative study in South India

**DOI:** 10.1186/s12884-023-06123-1

**Published:** 2023-11-21

**Authors:** Athira Sreenivas, Teddy Andrews Jaihind Jothikaran, Leslie Lewis, Mary Mathew

**Affiliations:** 1 Department of Pathology, Centre for Foetal and Perinatal Pathology, Kasturba Medical College, Manipal Academy of Higher Education, Manipal, Karnataka India; 2 Department of Social and Health Innovation, Prasanna School of Public Health, Manipal Academy of Higher Education, Manipal, Karnataka India; 3 Department of Pediatrics, Kasturba Medical College, Manipal Academy of Higher Education, Manipal, Karnataka India

**Keywords:** Community, Neonate, Perceptions, Postmortem, South India

## Abstract

**Background:**

Postmortem examination is the gold standard for establishing the cause of death. Minimally Invasive Tissue Sampling (MITS) of organs is a novel approach that can be used as an alternative to postmortem examination. In this study, the community perceptions in two states in South India towards neonatal postmortem and the acceptance towards the MITS technique is studied.

**Methods:**

In-depth interviews were conducted among the participants from Kerala and Karnataka to understand the perception towards postmortem and the acceptance of the novel MITS technique. The interviews were audio recorded, and a thematic analysis was done to identify the overarching themes and codes.

**Results:**

The knowledge and attitude of participants on conventional and MITS postmortem techniques, reasons for refusal, and the need for raising awareness were identified in the study. Participants favored the MITS techniques as it was less disfiguring and less time-consuming. The major concerns for refusal of conventional postmortem were that the procedure was disfiguring, time-consuming, and caused emotional stress to the parents.

**Conclusions:**

Participants favored the MITS approach over conventional postmortem as it caused less disfigurement and was conducive to the religious practice of burial of the body.

## Background

The average global neonatal mortality rate (NMR) is 18 deaths per 1,000 live births (2021) with South Asia accounting for 23 deaths per 1000 live births and 36% of the global newborn deaths. The NMR in India (2021) ranges between 17.1 to 21.4 [1]. Conventional diagnostic autopsy (CDA) is the gold standard for determining the cause of death in neonates. However, there is a global decline in neonatal postmortem due to lack of resources and knowledge regarding the procedure. In low-middle-income countries this information is obtained through verbal autopsies, which may not be sufficient to confirm the cause of death. There is no documented annual data on neonatal or infant autopsies in India, and verbal autopsies to ascertain the cause of death are not accurate [2]. There is lack of information regarding the cause of death in low-income countries; hence, there is a need to encourage neonatal or infant autopsies in India as this aids in establishing the cause of death, identifying relevant information that will help in counseling the parents and managing future pregnancies, as well as providing quality checks for antemortem diagnosis [3, 4]. In the Northern and West coast of India, the major reasons for refusal of autopsy in neonates are the attitude of the healthcare professionals, religious considerations, emotional distress of the parents, difficulty in obtaining consent, influence of community members, and economic liabilities [5–7]. Currently, there is no literature available in the South Indian context regarding community perceptions of conventional diagnostic autopsy. An alternative novel method to CDA is Minimally Invasive Tissue Sampling (MITS) of organs to identify the cause of death (CoD), and it is helpful in low-resource settings where CDA is refused [8–17]. This technique was developed by the Barcelona Institute for Global Health (ISGlobal) in 2013, and is a low-cost method done by inserting a specialized needle in targeted organs and obtaining a biopsy, which is used for diagnosis [18, 19]. The feasibility of this technique can bring early closure to parents of the neonate and prevent similar deaths in subsequent pregnancies [5, 20, 21]. We undertook this study to understand the knowledge and perceptions of the community regarding conventional diagnostic autopsy and MITS and to encourage the acceptance of this new technique as an alternative to establish the cause of death.

## Methods

### Aim

The aim of this study was to explore the community perceptions and barriers of postmortem examination and MITS in neonates.

### Study design

We approached this study with an exploratory qualitative research design to comprehend the perceptions of the community towards neonatal conventional autopsy and the acceptance of the MITS technique as a substitute.

### Study setting

This study was conducted in two states in South India, i.e., Karnataka and Kerala. Participants from the panchayats of Mala, Karkala Taluk, and Udupi district, Karnataka, and Peravoor panchayat, Iritty Taluk, Kannur district Kerala were included in the study. These two states were chosen due to the different infant mortality rates. The Infant Mortality Rate (IMR) recorded in Kerala is 6 and in Karnataka 21 per 1000 live births in 2019 [22].

### Participant recruitment

Married adult participants from both genders who have children or experienced miscarriages or abortion from Mala Panchayat, Karkala Taluk, Udupi District, Karnataka, and Peravoor Panchayat, Iritty Taluk, Kannur District, Kerala were selected through purposive sampling and snowballing technique for in-depth interview. A written consent was obtained from the participants. The ethnicity of the participants from both states were the same, but their social, linguistic, and cultural backgrounds were different. Permission from the respective Panchayats were obtained before the commencement of the study. Individuals from various women’s group, school teachers, and daily wage workers who met the inclusion criteria were included. The study included thirty-two in-depth face-to-face interviews with ten men and twenty-two women. Single adults and couples without children were excluded from the study (Table 1).

**Table 1:** Participant demographic details

Participants	Karnataka	Kerala
**Gender**		
Male	3	9
Female	13	7
**Age**		
30–39	5	2
40–49	7	3
50–59	4	5
60–69	0	4
70–79	0	2
**Education**		
Primary grad	4	2
High school grad	4	5
Graduate	5	4
Graduate and above	3	5
**Occupation**		
Daily wage	6	4
Government employee	5	6
Private sector employee	4	3
Unemployed	3	3
**Religion**		
Christian	0	8
Hindu	16	7
Muslim	0	1

### Ethical consideration

This study was approved by the Institutional Ethics Committee (IEC 384/2020). The participants were informed verbally in their native language about the strategy and purpose of the interview. Each participant received a printed version of the participant information sheet and an informed consent form, which included permission for an audio recording of the interview.

### Data collection and analysis

The data was collected from May 2021 to November 2021. Each interview was approximately 30–45 min. In order to understand the participant's perspective on autopsy, a semi-structured interview guide with probes was developed using existing literature. Knowledge and attitude towards postmortem examination, and awareness about MITS were included in the interview guide. Participants were asked about their perspectives on postmortem in general and then neonates in particular before the interviewer introduced the topic of the MITS technique and noted their views. Detailed field notes were taken during the interviews. The interviews were conducted in Kannada and Malayalam languages, later transcribed verbatim, and translated into English by the researcher, followed by crosschecking by the supervisor. The translated data was entered into Atlas ti.08 software for analysis. The transcripts were read and re-read multiple times and were analyzed thematically by generating codes, subthemes, and themes, which led to the final themes [23, 24].

## Results

The participants’ views and perceptions on postmortem examination and the MITS technique in Kerala and Karnataka were explored in this study. The interview commenced by gathering knowledge of the participant’s views regarding the postmortem technique, their concerns regarding neonatal postmortem, reasons for refusal, and contributing factors. After obtaining their attitudes and knowledge towards postmortem, the discussion on the novel method of postmortem examination—MITS technique was introduced in their native local language by showing the pictures of the needle and explaining the technique of obtaining tissue from the body and the participants’ opinions were recorded. Participants favored MITS as an alternative to conventional diagnostic postmortem examination as the technique is less disfiguring and would reduce emotional stress associated with the death of a baby but at the same time enabling them to know the cause of death. Five themes were derived on the basis of the participant’s responses (1) Perception on postmortem examination (2) Reasons for refusing postmortem (3) Knowledge regarding the MITS technique (4) Advantages perceived by the participants on the MITS technique (5) Need for awareness programs.

### Theme 1: perception on postmortem examination

#### Knowledge regarding postmortem examination

Although all the participants were familiar with the word “postmortem,” there were different opinions regarding the technique. Most of the participants expressed that postmortem examination is a procedure used to know the cause of death or in suspicious cases. Additionally, they found that postmortem could be beneficial for managing future pregnancies and academic purposes but was not useful in newborns.

*Teacher, 57, Female, Karnataka*

“*If it is a suspicious death, postmortem will be done…it will benefit. For a small baby I don’t think postmortem will help. For all deaths we cannot consent for autopsy. It depends on the type of death like unnatural or suspicious ones. And for small babies I don’t think there is a need.”*

Two participants stated that postmortem is a regular check for internal organs and can be used for donation purposes.

*Housewife, 39, Female, Kerala*

*“Organs will be used for donation purposes and postmortem is used to check the internal organs and for donation.”*

One participant stressed that when postmortem was done on people who had government jobs, it would enable their heirs to obtain placement in government service.

*Teacher, 59, Male, Karnataka*

*“It is mandatory to perform a postmortem if a government employee dies when they are in job. Then only their children will get their job, their provident fund all the insurance money. But the cause of death should be known in such cases postmortem helps.”*

Most of the participants perceived postmortem as a technique to know the cause of death in suspicious cases mainly, in adults, and felt it was not beneficial in children. They also acknowledged that postmortem can be used to educate medical students, manage future pregnancies and, donation of organs.

### Theme 2: reasons for refusing postmortem

#### Disfigurement and emotional stress

The primary reason for refusing an autopsy was the fear of disfigurement to the body. Another reason for refusal by parents and relatives was that the death of the baby would cause mental trauma, which would be compounded by doing a postmortem.

*Daily wager, 39, Female, Karnataka*

*“It must be difficult for them to the see their child disfigured after the procedure. Full body and face will be cut right? So no parents will be able to see their own child in such a situation”*

*Housewife, 59, Female, Kerala*

*“The parents will be deeply hurt… they do not want their baby to be cut open and bleed like an animal... postmortem in a child is irrelevant in society…once the baby has died, there is no point in doing an autopsy.”*

#### Time-consuming nature of the postmortem

Another concern was the time taken to return the body back to the family.

Clerk, 51, Male, Karnataka

*“I have gone for few postmortem, have waited a long time outside the room to get the body. It takes days to get the body back to the family.”*

#### Disrespect to the deceased and personnel conducting postmortem

Apart from disfigurement, few participants voiced their anxieties about the deceased's body being treated with disrespect and the procedure being carried out in an unprofessional manner by unqualified people. The participants differed in their opinion regarding the personnel conducting the postmortem. Few believed that postmortem was carried out only by government officials while others were of the opinion that common people who were brave enough would be chosen to conduct the postmortem by offering them alcohol. Doctors were only available to examine the body. They were concerned about the unprofessional manner of handling the deceased body, especially when performed by unqualified people.

*Librarian 51, Male, Kerala*

*“Doctors will not show interest to do a post-mortem, and it was done by other people where they will get alcohol and food to chop the skull…. the body taken will be laid on floor and nobody will care of the body and they will take an axe and hammer to break the head and the body… it will start to smell, later these hired people will do it and doctors will come and just investigate. And the rest of the things will be put back into the body in a very bad shape and re-stitched and packed”.*

#### Restriction to practice last rites following postmortem

Restriction in performing last rites on the dead person for whom a postmortem was done was an important concern expressed by the participants. A few participants, especially in Karnataka, said that "bathing the deceased," which is one of the principal after-death rituals, cannot be conducted if a postmortem is performed as the body cannot be unwrapped to give a ritual bath as there will be a lot of blood. A few participants also stated that if a post-mortem is performed, the parents will be restricted from touching the dead baby, and the physical and emotional depth of connecting with the baby will be reduced.

*Teacher, 59, Male, Karnataka*

*“There is ritual like taking bath for the body of deceased, so if postmortem is done, there will be presence of blood here and there, hence only face and feet can only be cleaned with water. If this is not done, later in society and family this will be a talk …they will be worried what others think.”*

Social worker, 42, Female, Karnataka

“At that moment before the funeral, both the father and mother will touch the baby to show their love… the last gesture of love due to her emotions...but if postmortem is done there will be restrictions to touch, kiss and hug the baby”.

#### Unaware of the benefits of doing postmortem

Some respondents brought up the issue of ignorance regarding the procedure and the lack of education leading to societal misunderstanding on the advantages and techniques of postmortem.

*Teacher, 55, Male, Kerala*

*“Parents will be unaware of the social protection and necessity of an autopsy. Some parents are unaware of it. The two basic justifications for a refusal that I observe is a result of an emotional attachment, and the other is brought on by a misunderstanding of the need for postmortem.”*

#### To restrain rumors in the society

Additionally, they expressed that if an autopsy is performed, rumors regarding the deceased’s cause of death and unnecessary talk would spread among the community.

*Retired civil police, 71, Male, Kerala*

*“Also, people will know, there will be a talk among the society about the post-mortem, they might discuss for what purpose the autopsy is being done, etc.”*

**Fig. 1:**
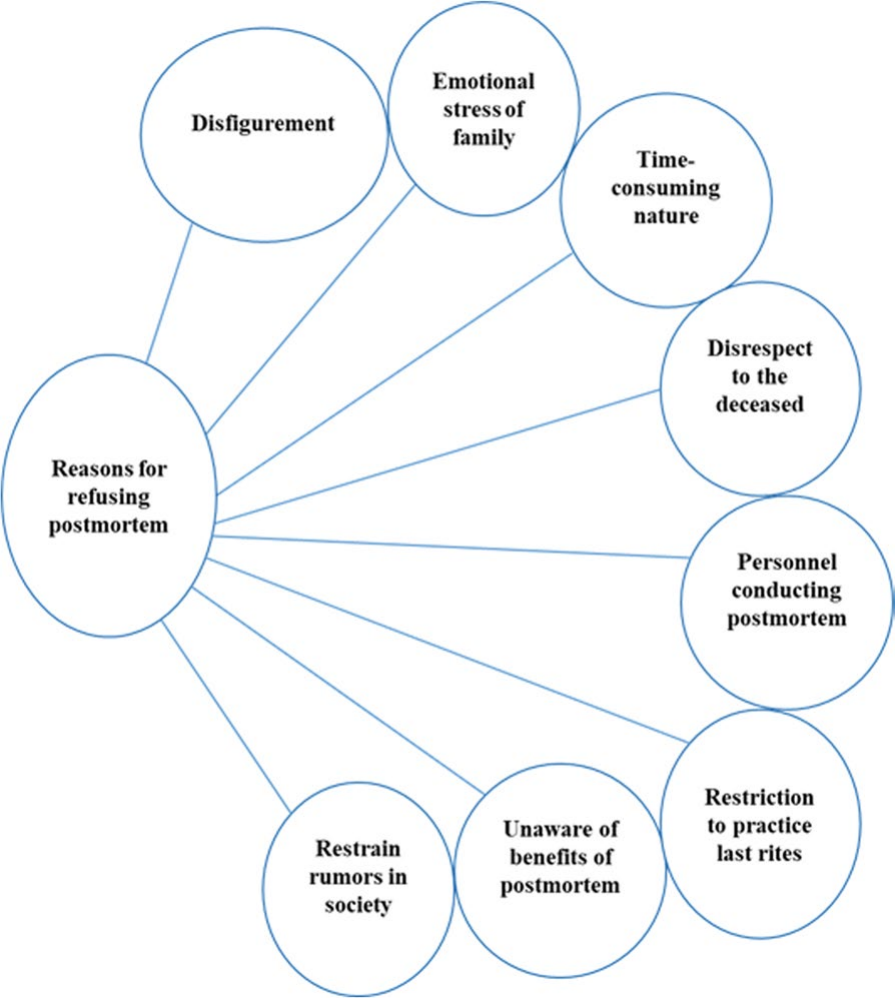
Reasons for refusing postmortem

The reservations with regard to postmortem for babies, according to participants, were the fear of disfigurement, emotional stress, disrespect to the body, delay in returning the body, awareness of the benefits of postmortem, and societal rumors (Fig. 1). Disfigurement of the body and its emotional impact of the death on parents were the over-arching concerns addressed by the participants.

### Theme 3: knowledge regarding the MITS technique

Although the word postmortem was familiar, the MITS technique of autopsy was not known to the majority of the participants. Except for three participants, the majority of participants were less informed or unaware of the MITS approach.

*Self-employed, 43, Female, Karnataka*

*“No, I am not aware about MITS.”*

### Theme 4: advantages perceived by the participants on the MITS technique

#### Less disfigurement

After listening to the knowledge shared by the researchers about MITS technique, the participants felt the MITS was a useful technique to replace the conventional autopsy. Most of the participants said that as this technique was less disfiguring compared to the conventional postmortem, this procedure would be helpful.

*Housewife, 52, Female, Kerala*

*“It is a nightmare to say that a baby’s body will be cut open. If we can investigate the cause of death using a needle, then it will be a great relief also we can avoid the disfigurement.”*

#### Less time-consuming

Compared to conventional autopsy, participants emphasized that society will certainly benefit from MITS as it is a less time-consuming procedure. They added that as the waiting period outside the mortuary is very painful and traumatic, this can be reduced if MITS was performed instead.

*Cook, 39, Female, Karnataka*

*“I think that will definitely benefit. To know the cause of death. This will be easy and fast, I think. The release of the body to relatives will be easy.”*

#### Reduce emotional stress

An additional benefit of the MITS technique, as perceived by the participants, was that it would help to reduce the emotional trauma experienced by the parents and other family members.

*Daily wager, 49, Male, Kerala*

*“There will be no mental stress seeing then body after this new autopsy technique which will benefit the society”*

#### No delay in practicing religious rites for burial

The non-invasive nature of the MITS technique made the participants agree that with this technique it is easier to perform religious ceremonies compared to conventional autopsy.

*Daily wager, 48, Female, Karnataka*

*“I think the new technique will be better, in this technique the reason for death is also found and there is no body and face disfigurement and can practice the rituals including bathing the deceased without any trouble.”*

The advantages perceived by the participants regarding the MITS technique is less disfigurement, less time-consuming, reduce emotional stress and no delay in practicing the last rites. They believed seeing less disfigurement would ease the emotional burden on parents when receiving the body back, especially in the case of children. Additionally, MITS offers early diagnosis, respect for rituals and last rites, and early return of the body.

### Theme 5: need for awareness programs

After understanding the advantages of MITS, a few participants stressed the need for outreach programs to be conducted in the community to inform people about the advantages of MITS. They stressed that MITS would be warmly welcomed if any awareness programming will be done. Additionally, these types of programs will help the public to understand better what is being done to the deceased body, reduce emotional stress, and negate the fear of postmortem.

*Teacher, 70, Female, Kerala*

*“We are ready to know the cause of death using this method. My opinion is that before implementing such a technique, try to make people aware of the novel technique. Give awareness classes to the people. Also, the fear in everyone hearing postmortem can also be reduced to a limit.”*

## Discussion

This study highlights the knowledge, perceptions, and attitudes of neonatal postmortem among people in Karnataka and Kerala. It also offers the major reasons for denying autopsy as well as the perspectives on the MITS procedure.

We identified some major concerns in CDA that are consistent with other studies, including improper handling, disrespect to the deceased's body, delay in returning the body, use for organs donation, and restrictions in conducting burial rituals [25, 26]. One of the main reasons postmortems were rejected was the fear of mutilation, especially in babies. Participants in this study stressed that it was meaningless to do a postmortem on a baby and it is essential only if the cause of death is unknown. Most of the participants believed that postmortem was performed only when there was a suspicion of the death of a child, which was similar to a study conducted in Vietnam [27]. Adverse media attention especially, in retaining organs, is another reason for the refusal of autopsy [28]. Studies across the world also find similar reasons for refusing autopsy, which was observed in our study [29–32]. Age, sex, and occupation did not alter participants' opinions on postmortem examination; however, participants from the Hindu religion felt that performing customary burial rites for the deceased was more challenging in conventional postmortem. Participants from Karnataka were particularly concerned about the post-death rituals of a baby compared to the participants in Kerala. One specific concern raised by the participants was related to the customary Hindu religious burial ritual of offering a "bath" to the deceased baby, which may be socially questioned if CDA is performed as the bathing process involves unwrapping the body of the neonate. Similarly, religious concerns for the autopsied body were highlighted in a study conducted in Pakistan [26]. Participants of the Hindu community from both states have emphasized their strong commitment to fulfilling the mandatory religious customs. Another major reason for refusal among participants was the fear of spreading rumors if rituals were not performed after the postmortem. This concern highlights the importance of performing rituals and the impact it has on people's beliefs and perceptions. Lack of knowledge regarding the benefits of autopsy was found to be an important factor with regard to conducting autopsy in children. The participants' educational backgrounds played an important role in the perceptions regarding postmortem. Among the participants, 53% were graduates, and 47% were a mixture of high school and elementary school education. The majority of the participants were unaware of the advantages of postmortem and felt that it was not relevant in babies; however, only in questionable circumstances it can be performed. One participant, who was a medical nurse, was aware of the procedures and the implications of doing a postmortem. A survey found that 42% of bereaved parents' grief was exacerbated by autopsy, and the authors advise conducting an open conversation with the parents about the benefits of autopsy [33]. The participants in this study were receptive to the MITS approach compared to the traditional autopsy. They find that the MITS procedure is less disfiguring, takes less time and reduces the emotional burden on the parents and family members and has no delay in offering rituals to the deceased. As a result, performing MITS was more acceptable than a conventional autopsy by the participants. Similar findings have been shared in other studies from North India, Mozambique, and Rwanda [5, 16, 21]. The participants find the MITS approach beneficial and conducive to religious rites performed after death which is not possible if a conventional autopsy is conducted. Few studies share similar findings about the religious concerns and delay of funerals due to CDA [29, 34, 35]. This highlights the necessity for awareness in society, regardless of a person's age, sex, level of education, profession, or faith. Our study recommends the need for increasing public awareness regarding the benefits of postmortem and the MITS technique as a feasible option in neonates as stressed by a few participants. This has also been recommended by other authors who felt the need to enhance public knowledge and eradicate preconceived notions about autopsy [36–38].

## Conclusion

This study examines the attitudes and beliefs concerning the practice of postmortem on neonates in Kerala and Karnataka. Fear of disfigurement, misinformation regarding the procedure, delay in obtaining the deceased body following the procedure, emotional distress, and inability to complete religious burial rites are major reasons for the rejection of postmortem. According to the findings of this study, educating and raising public awareness of the unique MITS approach and its significance and societal implications would assist the public in gaining understanding and lead to greater acceptance of the technique. The results of this study can help health-care workers to be sensitive to community concerns and plan education programs to increase the acceptance of autopsies in neonates. Additionally, establishing the cause of death in neonates and estimating the neonatal mortality rate can aid in distributing healthcare resources effectively.

### Strengths of the study

To the best of our knowledge, this is the first study to explore the perceptions of postmortem examination from two states in South India with different neonatal mortality rates. The study could identify the general perceptions of the community towards postmortem examination and MITS in neonates and the causes for refusal of postmortem in these communities.

### Limitations of the study

The limitation of this study was that the majority of the participants were from the Hindu community and there were only a few participants from the minority groups; hence, a contextualized perception could not be obtained.

## Data Availability

The datasets used and /or analysed during the current study are available from corresponding author on reasonable request.

## Abbreviations

CDA: Conventional Diagnostic Autopsy
CoD: Cause of Death
IMR: Infant Mortality Rate
MITS: Minimally Invasive Tissue Sampling
NMR: Neonatal Mortality Rate
